# Estimation of Coronavirus Disease Case-Fatality Risk in Real Time

**DOI:** 10.3201/eid2608.201096

**Published:** 2020-08

**Authors:** Yang Ge, Shengzhi Sun

**Affiliations:** The University of Georgia, Athens, Georgia, USA (Y. Ge);; Boston University School of Public Health, Boston, Massachusetts, USA (S. Sun)

**Keywords:** respiratory infections, severe acute respiratory syndrome coronavirus 2, SARS-CoV-2, SARS, COVID-19, 2019 novel coronavirus disease, coronavirus disease, zoonoses, viruses, coronavirus

## Abstract

We ran a simulation comparing 3 methods to calculate case-fatality risk for coronavirus disease using parameters described in previous studies. Case-fatality risk calculated from these methods all are biased at the early stage of the epidemic. When comparing real-time case-fatality risk, the current trajectory of the epidemic should be considered.

We read with interest the research letter on estimating case-fatality risk for coronavirus disease (COVID-19) by Wilson, et al. (*1*). In their analyses, the authors estimated the case-fatality risk adjusted to a fixed lag time to death. They acknowledged that the calculated adjusted case-fatality risk (aCFR) might be influenced by residual uncertainties from undiagnosed mild COVID-19 cases and a shortage of medical resources. However, we believe the time-varying number of cumulative cases and deaths also should be considered in the epidemic profile.

Because of the exponential growth curve of the COVID-19 outbreak, the numbers of cumulative cases and cumulative deaths have been relatively close to each other in the early stages of the outbreak, leading to a much higher aCFR. As the outbreak progresses, the ratio of the cumulative cases and deaths declines, which reduces the aCFR. Thus, a higher aCFR does not necessarily indicate increased disease severity.

To test our hypothesis, we performed a simulation study by using a susceptible-infectious-recovered–death model and parameters set according to prior studies. We set the infectious period as 10 days (*2*); case-fatality risk as 3% (*3*); basic reproductive ratio (R_0_) as 2.5 (*4*); recovery rate as 1/13 day (*5*), that is, 13 days from illness onset to recovery; and the population size as 1 million. We compared crude case-fatality risk, aCFR per Wilson et al.’s method, and aCFR per Mizumoto et al.’s method (*6*). Although the case-fatality risk calculated from these methods all are biased at the early stage of the epidemic, case-fatality risk calculated from Mizumoto et al.’s method was closer to the true case-fatality risk of 3% (Figure).

**Figure:**
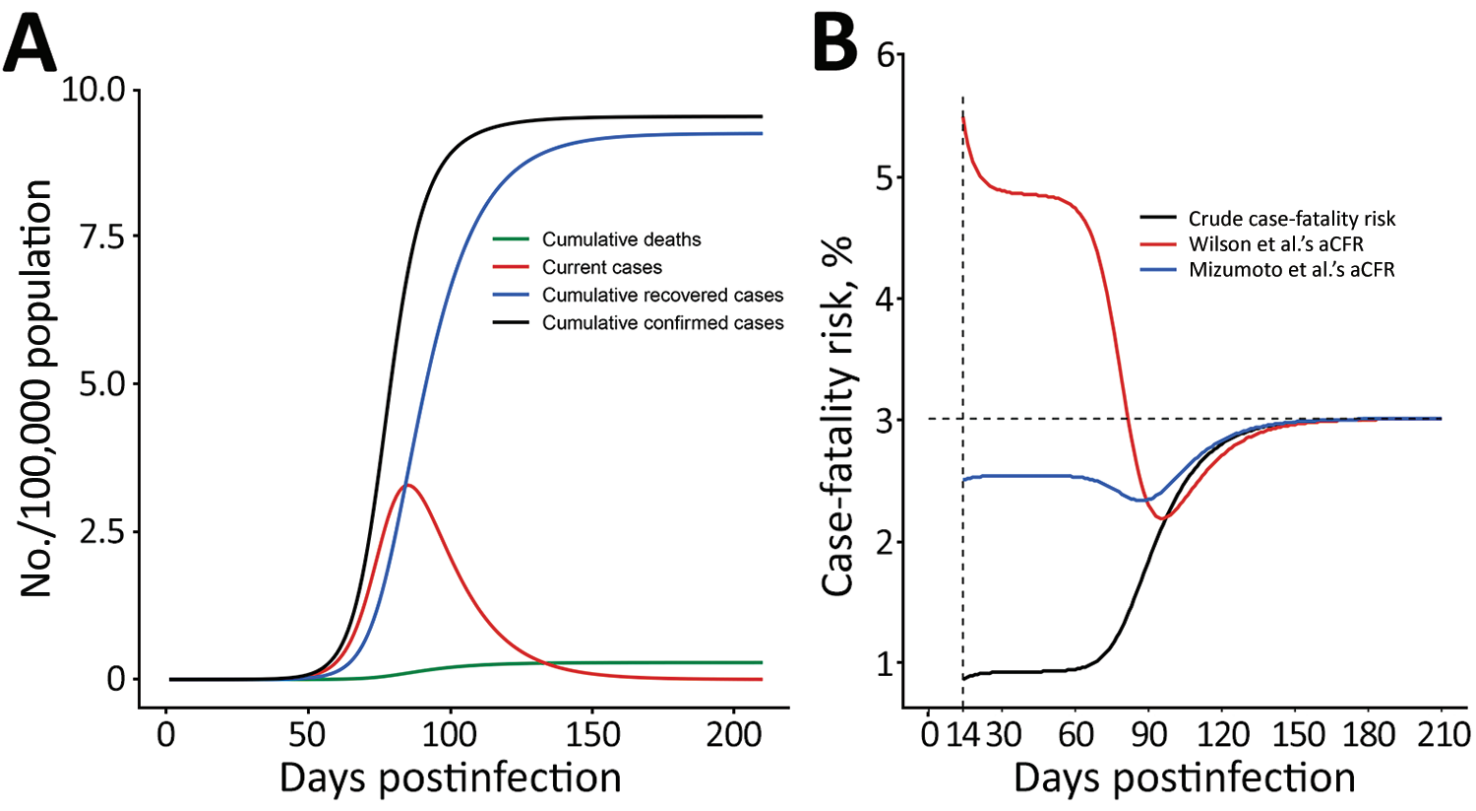
Progression of coronavirus disease outbreak and changes in the case-fatality risk by crude and adjusted rates. Crude case-fatality risk is the cumulative number of deaths on a given day divided by the cumulative number of cases on the same day. We set the infectious period as 10 days (*2*); case-fatality risk as 3% (*3*); basic reproductive ratio (R_0_) as 2.5 (*4*); recovery rate as 1/13 day (*5*), that is, 13 days from illness onset to recovery; and the population size as 1 million. A) Changes in the number of subpopulations over time after the first infection. B) Changes in crude case-fatality risk after 13th day of exposure and aCFR calculated by using Wilson et al.’s method (*1*) and by using Mizumoto et al.’s method (*6*). aCFR, adjusted case-fatality risk.

In conclusion, we recommend the Mizumoto et al. method (*6*) to calculate aCFR in real time. When comparing real-time estimation of the case-fatality risk across countries and regions, our results indicate that the current trajectory of the epidemic should be considered, particularly if the epidemic is still in its early growth phase.
